# Huntington's Disease: Two-Year Observational Follow-Up of Executive Function Evaluation with CNS Vital Signs Test in an Adult Patient

**DOI:** 10.1155/2011/385894

**Published:** 2011-12-04

**Authors:** Anna Lucia Spear King, Alexandre Martins Valença, Adriana Cardoso de Oliveira e Silva, Ana Claudia Cerqueira, Lígia Maria Chaves Ferraz, Antonio Egidio Nardi

**Affiliations:** ^1^Laboratory of Panic and Respiration (LABPR), Instituto de Psiquiatria (IPUB), Universidade Federal do Rio de Janeiro (UFRJ), RJ, Brazil; ^2^National Institute of Science Technology Translational Medicine (INCT-TM), Brazil; ^3^Instituto de Psiquiatria (IPUB), Centro de Ciências da Saúde (CCS), Universidade Federal do Rio de Janeiro (UFRJ), Av. Venceslau Brás, 71 Praia Vermelha, Cep 22290-140, Rio de Janeiro, RJ, Brazil; ^4^Department of Psychiatry and Mental Health, Universidade Federal Fluminense (UFF), Niterói, RJ, Brazil; ^5^Severino Sombra University (USS), Vassouras, RJ, Brazil; ^6^Laboratory of Thanatology and Psychometrics, Universidade Federal Fluminense (UFF), Niterói, RJ, Brazil; ^7^Rio de Janeiro Brazil Association of Huntington, Brazil; ^8^Medical School and Institute of Psychiatry, Universidade Federal do Rio de Janeiro (UFRJ), Brazil

## Abstract

Huntington's disease (HD) is a genetic, degenerative, and progressive central nervous system disease. It is characterized by motor abnormalities and cognitive and psychiatric symptoms. *Objective*. To describe the precise degree of clinical severity of patients with HD through a new neurocognitive assessment. *Methods*. Unprecedented battery of computerized tests, CNSVS (Central Nervous System Vital Signs), was applied at three different moments in 2008, 2009, and 2010. The accurate and reliable CNSVS objectively provided the cognitive state of patients and allowed for the evaluation of disease progression. *Case Report*. P., 26, female, without any medication, with normal psychomotor development is a parent carrier of HD. In 2008, she was diagnosed with HD in accordance with the Medical Genetics Laboratories. *Conclusion*. The tests may be useful to reveal the exact measure of the current evolutionary stage of HD patients, allowing for more efficient planning of treatment and future procedures, such as the medication, therapy, and physical activity to be administered.

## 1. Introduction

 Huntington's disease [1] (HD) is a genetic progressive heredodegenerative disease of the central nervous system (CNS). It is characterized by motor abnormalities and cognitive and psychiatric symptoms. The motor abnormalities may include muscle contractions, involuntary movements, loss of balance, poor coordination, speech difficulties, and excessive movements in stressful situations [1]. There is cognitive difficulty in organizing thoughts to handle new situations, tasks, loss of memory, and concentration. Psychiatric disorders include depression, irritability, anxiety, and behavioral changes [1].

 The first studies analyzed the inheritance and identified families from New England [2]. HD is related to the IT 15 gene mutation [3], which encodes the protein Huntingtin (HTT).

 The mean age of onset of HD varies between 35 and 45. If the age of the patient is 50 years or older (25% of cases), HD is considered to be “late-onset HD”, and before age 20 (approximately 10% of cases), it is defined as “juvenile HD”, which is the form that the case study patient has.

 George Huntington [4] published his paper “On Chorea” that described the familial form of the disorder referred to as “hereditary chorea”, which is a type of involuntary movement. HD occurs in all races, both sexes, and in the western population, with 30 to 70 individuals per million being affected. Using the differential diagnosis, it is not difficult to differentiate the conditions of many other HD choreas. They must observe the mode of inheritance [4], clinical course, prognosis, or findings in laboratory tests.

Diseases of the brain often affect an individual's behavior, including the impairment of cognitive skills and the production of neuropsychiatry symptoms. This report presents a neurocognitive assessment through a case study of HD. It uses a computerized neurocognitive test battery known as Central Nervous System Vital Signs (CNSVS) [5] that was applied in 2008, 2009, and 2010, with an interval of one year between each assessment, in a patient with HD who was not under any treatment (according to the patient's own decision).

The CNSVS was validated [6] reliable and suitable for use as a screening tool that measures speed and accuracy of basic mental functions. The test battery consisted of seven CNSVS tests: (1) visual memory (memorize 15 words and recognize them at the end); (2) verbal memory (memorize 15 geometric figures and recognize them at the end); (3) symbols (fill in the blank spaces with the symbol corresponding to a particular situation required in the proposed model); (4) warning (the computer proposes issues: color and shape or the individual should use the keyboards on the right or left to find the relevant answers); (5) perception of emotions (the individual must match facial expressions with emotions described below the figure proposed); (6) reasoning (various geometric figures are proposed to find associations and/or coordination among them); (7) recall of the first two tests, verbal memory and visual memory.

The results of the CNSVS objectively provided the cognitive state of patients and allowed the determination of the progression of the disease. It may assist the physician in clarifying the diagnosis and may be used periodically to observe the emergence of deficits in executive functions [7].

 Executive function [7] is a neuropsychological concept that applies to the cognitive process responsible for the execution and planning of activities including tasks, working memory, sustained attention, and inhibition of impulses. In addition, the memory's verbal, visual and composite processing speed, executive function, and sustained attention were assessed. People with HD may show changes in executive functions and possible functional manifestation of the brain and the onset of new symptoms [7].

 The aim of this case report was to describe the degree of clinical severity of patients with HD through neurocognitive assessment with an unprecedented battery of computerized tests, CNSVS, to provide the cognitive state of patients and allow for the evaluation of disease progression.

## 2. Case Report

P. is a 26-year-old female who is single, college graduate, and currently does not work. She lives with her mother and stepfather; her birth carried no complications, was breastfed up to 8 months of age, experienced her menarche at 12 years, began to be sexually active at the age of 16, and has normal psychomotor development.

 P. was diagnosed with HD in 2008 according to the Medical Genetics Laboratories [8] detected through a blood sample. The father carrier of HD died of medical complications at 40 years and had three more siblings with HD.

 In May 2008, when the patient was 25 years old, a genetic test [8] was conducted with a sample of blood. The result showed a mutation detected. Repeats Allele I: 58 and Allele II: 17.

 According to the DNA [8], one expanded allele of 58 repeats was detected and the other allele size was within the normal range for the laboratory. Thus, these results indicate that the individual has the common genetic alteration found in HD. Genetic counseling was recommended for at-risk family members.

 Analysis of the DNA from this individual has been used to study the size(s) of the CAG [9] (trinucleotide repeat) repeat region of the Huntington gene. The normal range is considered to be up 28 copies of the repeat. Alleles with 27 repeats up to and including 35 repeats are considered to be normal mutable alleles. To date, symptoms of HD have not been reported in individuals with 35 or less repeats. Allele sizes of 36 up to and including 38 repeats are considered to be Huntington alleles with reduced penetrance. HD alleles (mutations) are 40 repeats and greater. The reported sizes for large expansions are approximations due to the precision of the size measurements using current technology. The genotype-phenotype correlations are not precise enough to be used clinically [9].

 From diagnosis until the present time, the patient did not use any medication for HD. The family was advised to seek medical treatment and did so by consulting several specialists. The patient decided not to start treatment with medication despite medical advice.

 At 25, P. began to present difficulties in dealing with new situations, such as performing daily tasks. Also, she displayed a lack of concentration, coordination, mood swings, and some involuntary movements.

 The patient sought treatment with complaints of depression and difficulty with attention and concentration. Then, P. was invited to participate in the study with CNSVS so that we could assess the degree of impairment in her executive functions. The patient signed the informed consent and was made aware of all procedures, and the study was approved by the Ethics Committee for Research of the Institute of Psychiatry (IPUB), Federal University of Rio de Janeiro (UFRJ).

## 3. Relevance and Originality

Clinicians and researchers, with the help of neurocognitive tests, accurately assessed the pathology of neurological patients and quantitatively measure the health of the higher functions of the brain and CNS.

## 4. Results

The absence of medication during the evaluation period allowed us to analyze the evolutionary aspects of their clinical and mental function. All tests were applied using the same psychologist, office environment, and evaluator, with the same level of cooperation from the patient.

Table 1 contains five levels of clinical severity from the CNSVS [5] tests, based on a database with more than 1900 subjects and aged between 8 and 90 years. The levels are classified as follows: Above: >110 (high function and high capacity); Average: 90–110 (normal function and normal capacity; Low Average: 80–90 (sight deficit and sight impairment); Low: 70–79 (moderate deficit and impairment possible); Very Low: <70 (deficit and impairment likely).

Table 2 contains the instrument panel regarding neurocognitive assessments 2008, 2009, and 2010. The test results of the CNSVS reported in Table 2 are that the patient did not achieve any results categorized as “Above” in the tests from 2008, 2009, and 2010, which indicates that she did not display high function or high capacity in the proposed tasks.

 As shown in Table 2, the patient's verbal and visual memory were “Average” in 2008. In 2009, the patient's composite and verbal memory had declined to “Low”, in 2010, the patient's composite and verbal memory were “Very Low”, representing injury unlike the results for visual memory, which only showed a moderate deficit.

 The patient's processing speed was “Very Low” in 2008 and improved to “Low” in 2009 but in 2010 was reduced to “Very Low”, showing deficits and probable injury.

 The patient's executive function remained “Average” in 2008, 2009, and 2010, showing normal function and capabilities. Her social acuity in 2008 was “Average”, and in 2009 and 2010, it was “Low Average” showing a slight deficit and slight impairment.

 In 2008 and 2009, the patient's ability to reason was already “Low” and decayed a bit in 2010 to “Very Low”, which indicates probable deficits and impairment. The patient's ability to sustain attention in 2008 was “Low Average” and in 2009 recovered to “Average” but returned in 2010 to “Low Average”, indicating a slight deficit and impairment. Similarly her working memory was “Low Average” in 2008 and recovered to “Average” in 2009 but returned in 2010 to “Low Average”, indicating a slight deficit and impairment.

## 5. Discussion

 Brain injuries [10] often produce the loss of cognitive skills, neuropsychiatry symptoms, and behavioral changes. The precise knowledge of the presence and characteristics of these manifestations may help in the diagnosis and neurological, psychological, and psychiatric management and care in patients with HD.

 In our case study, we observed a significant decrease of cognitive and social function in the patient studied, which contributed to a loss of social, occupational, and impairment in the quality of life. The CNSVS [5] may help clinicians to assess cognitive function of the individual and to accurately identify their symptoms, impairments, and comorbidities.

 Studies [11] on cognitive ability (thinking, judging, and memory) are necessary to further our knowledge of diseases such as HD, a degenerative disease with symptoms that hinder emotional growth and development during its progression and evolution due to a loss of cells in the basal ganglia [11].

 The possibility of a step-by-step follow-up is necessary because the onset of symptoms allows us [12] to more efficiently assign immediate therapies targeted to specific deficits and, therefore, offers a possible opportunity to slow the development of HD.

Only in recent decades have researchers [13] begun to study cognitive deficits and neurological disorder at the molecular level. What emerged from these studies [12] was how the cells within the CNS communicate with each other during the process of learning.

 Recall that our study had a limited time period (2008–2010) taking advantage of the period in which the patient did not use medication to not interfere in the results. We recommend a possible use of this tool (CNSVS) in long-term clinical trials and with larger samples and also compared with other cognitive tests. We know that Huntington's disease may have a broader course of 10–20 years.

 Consider it relevant to the use of computerized tests that allow to accurately assess the patient's reaction time to disease progression related to executive functions. That is, with this type of test, periodically we see the emergence or absence of deficits in these functions.

## 6. Conclusion

 The study of the degree of clinical severity through CNSVS may be very important for measuring cognitive disturbances [14, 15] in HD patients, helping the clinician to more effectively plan the treatment and procedures, such as pharmacological treatment, psychotherapy, and physical activity to be administered.

## Figures and Tables

**Table 1:** Clinical levels of severity (CNSVS). The CNSVS [5] contains five degrees of clinical severity based on a database with more than 1900 subjects, aged between 8 and 90 years.

Above	>110	High function and high capacity
Average	90–110	Normal function and normal capacity
Low Average	80–90	Sight deficit and sight impairment
Low	70–79	Moderate deficit and impairment possible
Very Low	<70	Deficit and impairment likely

**Table 2:** Dashboard neurocognitive. Test Results CNSVS the years 2008, 2009, and 2010.

Subject: P.	Percentile range									>74			25−74			9–24			2–8			<2		
Ages: 25, 26, 27	Standard score range									>109			90–109			80–89			70–79			<70		
Profile																								
Scores	Subject score			Standard score			Percentile			Above			Average			Low Average			Low			Very Low		
	08	09	10	08	09	10	08	09	10	08	09	10	08	09	10	08	09	10	08	09	10	08	09	10
Composite memory	** 102**	** 87**	** 79**	** 103 **	** 75**	** 59**	** 58**	** 5**	** 1**				** X**							**X**				**X**
Verbal memory	** 52 **	** 45**	** 42**	** 97**	** 71**	** 61**	** 42**	** 3**	** 1**				** X**							**X**				**X**
Visual memory	** 50 **	** 42**	** 37**	** 107**	** 85**	** 71**	** 68**	** 16**	** 3 **				** X**				**X**				**X**			
Processing speed	** 34**	** 45**	** 31**	** 62**	** 75**	** 58**	** 1**	** 5**	** 1**											**X**		**X**		**X**
Executive function	** 47 **	** 46**	** 44**	** 94**	** 93**	** 90**	** 34**	** 32**	** 25**				** X**	** X**	** X**									
Social acuity	** 7 **	** 5**	** 6**	** 92**	** 80**	** 86**	** 30**	** 9**	** 18**				** X**				**X**	**X**						
Reasoning	** 1 **	** 1**	** −1**	** 76**	** 76**	** 69**	** 5**	** 5**	** 2**										**X**	** X**				**X**
Sustained attention	** 22 **	** 27**	** 22**	** 88**	** 96**	** 88**	** 21**	** 40**	** 21**					** X**		** X**		**X**						
Working memory	** 5 **	** 9**	** 6**	** 81**	** 95**	** 84**	** 10**	** 37**	** 14**					** X**		** X**		** X**						
*Additional data	** 1 **	** 1 **	** 1**	** 2 **	** 2**	** 2 **	** 3 **	** 3**	** 3**	** 4**	4	4	** 4**	** 4 **	** 4 **	** 4 **	4	4	4	4	4	4	4	4

Obs. 2008 (08); 2009 (09); 2010 (10).

Reaction time (milliseconds). In 2008—1171; In 2009—1180; In 2010—1315.

Total test time: 2008 (20 : 44 min/sec); 2009 (22 : 24 min/sec); 2010 (21 : 48 min/sec).

*Additional data:

(1) Subject scores: computed from the raw score calculations using the data values of the individual subtests and are simply the number of correct responses, incorrect responses, and reaction times.

(2) Standard score: In CNSVS the average standard score of the group is 100 and standard deviation is 15.

(3) Scale of 1 to 100.

(4) Normative scores.
